# Changes in the Volatile Flavor Compounds and Quality Attributes of Tilapia Fillets Throughout the Drying Process

**DOI:** 10.3390/foods14193293

**Published:** 2025-09-23

**Authors:** Jun Li, Huan Xiang, Shuxian Hao, Lina Wei, Hui Huang, Ya Wei, Shengjun Chen, Yongqiang Zhao

**Affiliations:** 1 Guangdong Provincial Key Laboratory of Lingnan Specialty Food Science and Technology, Key Laboratory of Green Processing and Intelligent Manufacturing of Lingnan Specialty Food, Ministry of Agriculture and Rural, College of Light Industry and Food, Zhongkai University of Agriculture and Engineering, Guangzhou 510225, China; 2 Key Laboratory of Aquatic Product Processing, Ministry of Agriculture and Rural Affairs, National R&D Center for Aquatic Product Processing, South China Sea Fisheries Research Institute, Chinese Academy of Fishery Sciences, Guangzhou 510300, China; susan2001@163.com (S.H.); 13677811513@163.com (L.W.); huanghuigd@aliyun.com (H.H.); weiya@scsfri.ac.cn (Y.W.); chenshengjun@scsfri.ac.cn (S.C.); zhaoyq@scsfri.ac.cn (Y.Z.); 3 Key Laboratory of Efficient Utilization and Processing of Marine Fishery Resources of Hainan Province, Sanya Tropical Fisheries Research Institute, Sanya 572018, China

**Keywords:** ready-to-eat tilapia fillets, drying processing, quality, volatile flavor substances

## Abstract

The rising popularity of ready-to-eat self-heating sauerkraut fish necessitates a meticulous production process to ensure high-quality products. This study investigated the impact of processing stages on the quality of ready-to-eat tilapia fillets. The results showed that lipid oxidation, protein degradation, pH levels, and TBA concentrations increased during processing. GC-IMS analysis revealed 56 volatile compounds in tilapia fillets, with distinct compositions at different processing stages. The flavor profiles of tilapia fillets underwent significant changes during blanching and rehydration. The levels of aldehydes and alcohols notably increased, with the blanching group exhibiting the highest concentration of aldehydes, particularly saturated linear aldehydes such as hexanal, nonanal, octanal, and benzaldehyde, which play key roles in enhancing fish flavor. Conversely, the proportion of ketones decreased following heat treatment, which is a crucial factor in mitigating undesirable fishy odors. Therefore, the optimal method for preparing ready-to-eat tilapia fillets was salting pretreatment (1.5% salt and 3% propylene glycol) at 4 °C for 1 h, blanching at 100 °C for 1 min, pre-freezing at −40 °C for 12 h, and vacuum freeze-drying at −40 °C under 20 Pa for 18 h. Finally, the dried fish fillets were vacuum-sealed for storage. Principal Component Analysis (PCA) revealed that the combined variance explained by the first two principal components post-dimensionality reduction was 95%, serving as a primary indicator of the volatile flavor profile of the fish. The dried fillets were thoroughly verified using sensory evaluation. This specific formulation garnered the highest scores in sensory evaluations, resulting in superior aroma, color, and texture attributes for the self-heating fish product. The findings of this study offer a foundational framework for developing ready-to-eat tilapia fillets and other convenient food products in the future.

## 1. Introduction

A fast-paced lifestyle promotes the development of convenient food, which is a factory combination of traditional dishes and processed for direct consumption or simple re-heating. Self-heating food offers the convenience of portability and consumption, utilizing self-heating technology that eliminates the need for electricity, open flames, gas, or other heating equipment to heat food [1]. The reheating process is simple and rapid, facilitating the advancement of Chinese cuisine and convenience foods, while expanding the diversity of self-heating product offerings.

Tilapia (*Oreochromis niloticus*) is one of the most extensively cultivated freshwater fish globally and is valued by consumers for its balanced nutritional profile, which includes various unsaturated fatty acids and proteins [2]. However, its high moisture content presents challenges in terms of preservation [3]. Currently, tilapia products in the market are predominantly live fish and frozen fillets, which are less refined and processed than other fish products [4]. Dried fish fillets, canned fish, waxed products, and surimi products are the primary tilapia products that require extensive processing [5]. Currently, the market offers a limited selection of ready-to-eat tilapia products. Consequently, expanding the diversity of tilapia-based offerings has emerged as a significant research focus with promising potential applications. The rise in convenient self-heating instant meals has led to an increased demand for fish fillets in self-heating hot pots. Heat processing is one of the most common methods for preparing ready-to-eat products with an extended shelf life [6]. Drying is a commonly used storage method that can reduce moisture content and improve the flavor of ready-to-eat food [7]. For aquatic products, requirements of convenience and sensory quality need to be solved by investigation on the basis of food security. However, different thermal processing methods may result in protein denaturation, which affects protein properties and meat texture [8]. The water-soluble taste substances in tilapia fillets vary with different thermal processing methods (steaming, boiling, and air frying) [9]. Moreover, the heating treatment combined with the drying method might be suitable for making ready-to-eat fillets, although there are limited reports.

In the preliminary stage, our research group studied the effects of heat treatment and pickling pretreatment on the texture of tilapia. The results showed that blanching treatment improved the freeze-drying rate of fish, reduced the drying time required, and enhanced the rehydration properties and palatability of dried fish fillets. However, there is no information on the changes in volatile components during heating and drying. Volatile components affect the overall flavor of fish, which is an important indicator of fish flavor quality. Different degrees of chemical reactions occur during curing and heat treatment before drying, which affect the quality and flavor of the meat products. The curing process causes lipid and protein oxidation, which significantly affects meat quality and flavor [8]. NaCl promotes lipid and protein oxidation during processing, thereby affecting the texture, color, and flavor of the food [10]. Thermal processing promotes lipid oxidation to produce linear aldehydes, alcohols, and acids and affects the water-soluble flavor substances in fish [11]. Most dried products need to be rehydrated before consumption, and the reheating method and time affect their characteristic flavors [12]. The long steaming time and consumption of large amounts of flavor precursors resulted in the production of abundant volatile substances. However, there is limited understanding of the potential differences among various drying methods in terms of the formation of flavor-contributing volatile compounds in tilapia fillets.

Gas chromatography–ion mobility spectrometry (GC-IMS) is an advanced technique for the rapid detection of flavor compounds. It has the advantages of fast response, high sensitivity, and relatively low instrument cost and has been widely used in meat flavor analysis [13]. Li et al. [6] found that the characteristic flavor substances of chicken thighs smoked with different condiments were related and different in fingerprint information and principal component analysis.

In this context, the effects of different treatments, including pickling, blanching, freezing, and rehydration after drying, on the quality (texture properties, pH, and thiobarbituric acid (TBA)) and volatile components of tilapia (*Oreochromis mossambicus*) fillets were investigated. GC-IMS was used to compare the differences in volatile flavor substances in tilapia fillets subjected to different treatments. Principal component analysis (PCA) was used to differentiate between tilapia fillets subjected to different treatments. This study clarifies the impact of heating packs on the rehydration efficiency, heating performance, and product quality of freeze-dried tilapia, ultimately contributing to the development of self-heating tilapia products. This study aimed to provide a theoretical basis for the processing conditions of ready-to-eat tilapia fillets for commercial production of tilapia fish.

## 2. Materials and Methods

### 2.1. Materials and Reagents

Edible salt was purchased from the Guangdong Salt Industry Group (Guangzhou, China). Propylene glycol (food grade) was purchased from Meiyuan Biotechnology Co., Ltd. (Guangzhou, China). Analytically pure thiobarbituric acid (TBA) and trichloroacetic acid (TCA) were purchased from the Guangzhou Chemical Reagent Factory. A special food heat-source material pack (aluminum powder, calcium oxide, and sodium carbonate) was purchased from Fengtai New Material Technology Co., Ltd. (Beijing, China). Seasoned and pickled cabbage packs were purchased from Sichuan Tianwei Food Group Co., Ltd. (Chengdu, China).

### 2.2. Preparation of Ready-to-Eat Tilapia Fillets

#### 2.2.1. Operation Key Points

Forty-eight fresh tilapia (*O. niloticus*, average weight 500 ± 50 g) were purchased from Guangdong Huanqiu Aquatic Food Co., Ltd. (Huazhou, China) and rapidly transported to the laboratory. Fresh tilapia was initially sectioned into pieces measuring 3 cm × 3 cm × 1 cm (A, fresh tilapia fillets). The fillets were then subjected to a pickling process involving salt (1.5%) and propylene glycol (3%, mass fraction) at 4 °C for 1 h, followed by heating at 100 °C for 1 min (B, pickled). Subsequently, the blanched tilapia fillets were allowed to cool to room temperature and pre-frozen at −40 °C for 12 h (C). They were then subjected to vacuum freeze-drying (cold trap temperature: −70 °C, vacuum degree: 20 Pa) to produce dried products. Finally, the dried tilapia fillets were heated in a heating package (90 g) for 20 min to obtain the rehydrated product (D).

#### 2.2.2. Texture Measurement

The textural properties of the samples were assessed using a previously established method with some modifications [14]. The hardness, elasticity, and chewiness of fresh tilapia fillets (A), pickled and blanched tilapia fillets (steamed at 100 °C for 1 min, B), freeze-dried tilapia fillets (C), and rehydrated tilapia fillets (D) were evaluated using a texture analyzer (QTS-25; CNS Farnell, Borehamwood, UK) in six replicates. The cylindrical probe was designated as P/44, with a test speed of 1.0 mm/s for two cycles, a trigger force of 5.0 g, and a downward pressure of 10.0 N.

#### 2.2.3. Determination of pH

Tilapia fillets (2.0 g) were combined with 18 mL of distilled water, and the mixture was homogenized and centrifuged at 8000× *g* for 10 min. After filtration, the pH was determined using a pH meter [15].

#### 2.2.4. Measurement of Thiobarbituric Acid (TBA)

TBA was measured following the methodology outlined by Piranavatharsan et al. [16]. A sample weighing 1.0 g was mixed with 4 mL of distilled water and 5 mL of a pre-cooled TCA solution with a 20% mass fraction. The resulting mixture was homogenized and maintained on ice for one hour. Subsequently, the mixture was centrifuged at 8000× *g* at 4 °C for 10 min, and the supernatant was transferred to a 10 mL volumetric flask. The supernatant was then combined with 5 mL of a *TBA* solution (0.02 mol/L), heated in boiling water for 20 min, and allowed to cool to room temperature. Distilled water was used as the blank. Absorbance was measured using a UV spectrophotometer at A532 nm, and the results were expressed as mg/100 g of sample.

(1) TBA=A×7.8

#### 2.2.5. Analysis of Volatile Flavor Components

The analysis of volatile flavor components in tilapia fillets across different processing stages was conducted using a HS-GC-IMS instrument (FlavourSpec^®^ Flavor) from G.A.S (Dortmund, Germany). A five-gram portion of tilapia fillet was placed in a 20 mL headspace vial. The samples were then incubated at 80 °C for 20 min. Following incubation, 500 μL of the headspace was automatically injected through a heated syringe (85 °C) into the GC-IMS apparatus. This procedure was consistently applied to three parallel samples to ensure uniformity in processing. Gas chromatographic separation was performed at 60 °C on an MXT-5 capillary chromatographic column (15 mL × 0.53 mm, 1 μm). High-purity nitrogen was used as a drift gas at a flow rate of 150 mL/min and as a carrier gas with the following program: 2 mL/min for 2 min, linear increase to 100 mL/min over 18 min, and a total run time of 20 min. When subjected to an electric field and reverse drift gas, the samples advanced towards a Faraday disk for detection, facilitating secondary separation. The n-ketones C4-C9, sourced from Sinopharm Chemical Reagent Beijing Co., Ltd., Beijing, China, served as external standards for calculating the retention indices (RI) of each volatile compound. Mass spectrometry analysis was conducted in the electron collision mode at 70 eV and full scan mode, ranging from 29 to 550 Da. Each volatile compound was identified by referencing the NIST 17 mass spectrometry database, with further characterization through deconvolution using AMDIS software (http://amdis.net/, accessed on 29 July 2025). The percentage of the peak area represents the relative concentration of each flavor compound in the sample.

### 2.3. Influence of Heat Source Material Pack on the Quality of Dried Tilapia Fillets

#### 2.3.1. Effect of Heat Source Material Pack on the Heat Transfer of Dried Tilapia Fillets

The self-heating package was positioned at the base of the container, and the lid was closed. Subsequently, 500 mL of water was poured into the food compartment, and dried tilapia fillets (50 g) were added. A temperature probe was inserted into the center of the fish to assess the impact of the heating package on the core temperature of tilapia fillets.

#### 2.3.2. Effect of Heat Source Material Pack on the Rehydration Rate and Texture Changes in Dried Tilapia Fillets

The heating bag was placed at the bottom of the self-heating container, dried tilapia fillets were placed in the tank of the self-heating food box, 500 mL water was added, 90 g of the heating bag was rehydrated for 30 min, and the weight of the rehydrated sample was recorded every five minutes.

(2) $$Rehydrationrate/\%=\frac{m_{1}-m_{0}}{m_{1}}\times 100$$

The texture was determined as described in Section 2.2.2.

### 2.4. The Sensory Evaluation of Self-Heating Sauerkraut Fish

Freeze-dried tilapia fillets (50 g) were used to investigate the impact of varying quantities and reheating durations of sauerkraut and seasoning packets on the sensory evaluation of reheated sauerkraut fish. Self-heating sauerkraut fish was prepared using 80 g of pickled vegetables, 50 g of seasoning, and 50 g tilapia fillets. For sensory evaluation, a total of 10 evaluators, including five women and five men aged 22–28 years, formed the sensory evaluation group according to Chen et al. [17]. The recruitment and consent processes were conducted in accordance with the guidelines of the Declaration of Helsinki, thereby ensuring ethical compliance. The evaluators were trained and familiarized with the method of evaluating the self-heating sauerkraut fish. The data in this study were collected with the informed consent of all evaluators. All evaluators agreed to participate and use their information and were fully informed of how the data would be used in this study. The sensory evaluation focused on five aspects: odor, taste, color, texture, and overall acceptability based on a ten-point scale (Table S1). The sensory evaluation of the self-heating sauerkraut fish was performed in an odor-free room, and the evaluators rinsed their mouths with purified water before sample evaluation.

### 2.5. Data Analysis

SPSS software (version 17.0; SPSS Inc., Chicago, IL, USA) was used for one-way analysis of variance (ANOVA). All experiments were repeated three times, and the data are expressed as the mean ± SD. Significant differences between the data were defined as *p* < 0.05 and were evaluated using Tukey’s test. PCA was performed using the MetaboAnalyst software (6.0), according to Li et al. [18]. VOCs were analyzed using a laboratory analytical viewer and GC×IMS Library Search (FlavourSpec^®^ Flavor) and qualitatively analyzed using the NIST and IMS databases developed in the software. The signal intensity represents the height or the peak area.ng laboratory analytical viewer, reporter, gallery plot, and three-dimensional (3D) and two-dimensional (2D) fingerprint maps of the VOCs of tilapia fillets in different processing stages were constructed.

## 3. Results and Discussion

### 3.1. Quality of Tilapia Fillets in Different Processing Stages

Texture is a crucial sensory attribute that influences consumer acceptance and fish processing methods. The changes in texture characteristics, such as hardness, elasticity, and chewiness, are presented in Table 1. Compared to fresh tilapia fillets, the hardness and elasticity were significantly increased (*p* < 0.05) in the freeze-dried fillets. The chewiness of freeze-dried fillets (pickled with salt and propylene glycol before being freeze-dried) was significantly higher than that of fresh and rehydrated tilapia fillets (*p* < 0.05), indicating that salting effectively maintained the edible taste of fish, which is consistent with previous research showing that salt addition during blanching could change meat quality [19]. The primary reason for this phenomenon is the effect of NaCl on the structural organization and denaturation of fish myofibrillar proteins during curing. This results in a reduction in moisture content, alteration of protein hydration, and, consequently, a change in fish texture [20]. No significant differences were observed in the hardness, elasticity, or chewiness of the rehydrated fillets compared to those of the pickled and blanched tilapia fillets (*p* > 0.05).

To a certain degree, pH acts as an indicator of changes in the muscle quality of aquatic products and is frequently regarded as a critical measure for evaluating fish muscle quality. As shown in Table 1, fresh samples had the lowest pH, which increased significantly after blanching, freezing, and subsequent rehydration (*p* < 0.05). There was no significant change in pH during the pre-freezing process (*p* > 0.05), and fish in the rehydration group had the highest pH. Previous studies have also shown that the pH of fish increases significantly after heat treatment, and the increase in fish pH is related to protein hydrolysis. The pH of fish meat increases after heat treatment, primarily because of the high temperatures that cause destabilization of the protein structure and a decrease in the number of acidic groups within the protein [6]. Concurrently, rising temperatures accelerate the breakdown of fish meat proteins into amines, thereby increasing pH levels.

Fat undergoes hydrolysis and oxidation under heat and O_2_ conditions to produce malondialdehyde (MDA), which forms a stable complex with TBA. The TBA value is closely related to the degree of fat oxidation in aquatic products. Compared to fresh tilapia fillets (Table 1), the TBA value of dried tilapia increased significantly (*p* < 0.05). The TBA value of the rehydrated tilapia fillets was the highest, increasing from 0.39 ± 0.01 mg/100 g (fresh tilapia fillets) to 0.52 ± 0.01 mg/100 g (rehydrated tilapia fillets) after storage. The reason lies in the fact that tilapia meat comprises polyunsaturated fatty acids. These fatty acids are susceptible to temperature fluctuations, microbial activity, and oxygen exposure during processing, leading to increased fat oxidation. Sodium ions that penetrate the pickling process accelerate the fat oxidation. This may also result from the disruption of cellular membrane structural integrity and oxidative reactions between oxidants and unsaturated lipids [21]. Therefore, the TBA value of the fish increased significantly after pickling and blanching. Heating pretreatment promotes fat oxidation, producing various volatile flavor substances and promoting the formation of fish flavor. Studies have shown that heating promotes the oxidation of horse fat to produce aroma, and moderate lipid oxidation is conducive to the production of aromatic substances [22]. The maximum TBA value after rehydration was mainly due to the high temperature and long heating time, which aggravated the oxidation of fat and increased the malondialdehyde content, a derivative formed by the oxidative degradation of saturated fatty acids.

### 3.2. Fingerprint Analysis of Flavor Substances in Tilapia Fillets Under Different Processing Stages

**Figure 1:**
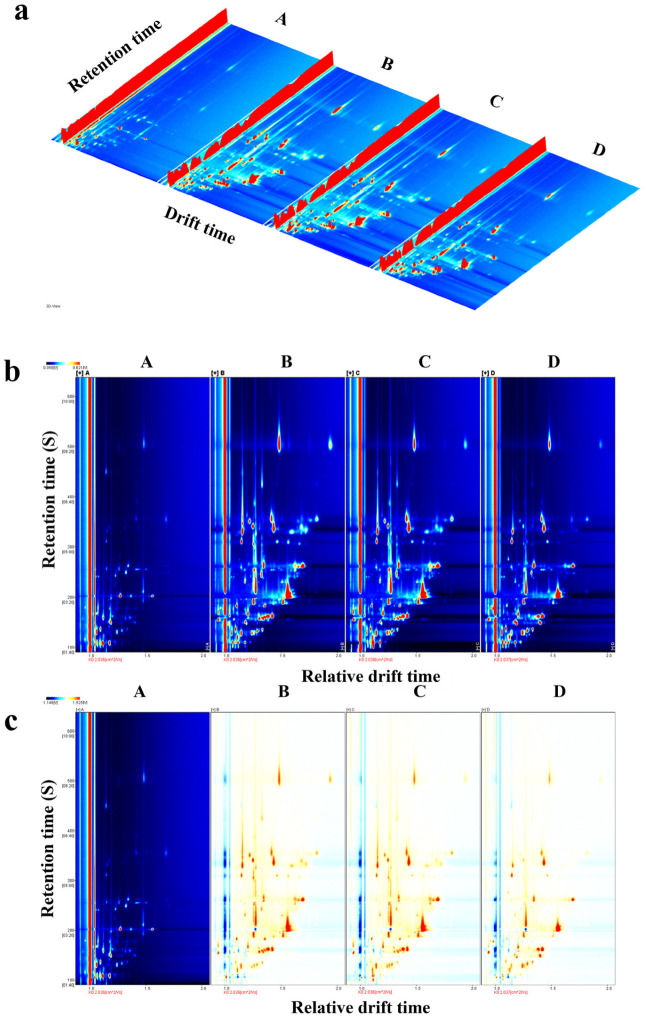
Fingerprint analysis of flavor substances in tilapia fillets at different processing stages. (**a**) Three-dimensional spectral profiles of volatile compounds. (**b**) Top-view spectral profiles of volatile compounds. (**c**) Comparative differential spectral profiles of volatile compounds. Note: (1) The background of the entire figure is blue, and the red vertical line at abscissa 1.0 is the rip peak (reactive ion peak, normalized). (2) The ordinate represents the retention time (s) for gas chromatography, and the abscissa represents the ion migration time (normalization). (3) Each point on both sides of the rip peak represents a volatile organic compound (VOC). The color represents the concentration of the substance; white indicates a low concentration, red indicates a high concentration, and the darker the color, the greater the concentration.

The changes in the types and concentrations of volatile flavor substances during tilapia fillet processing were monitored using GC-IMS. Variations in VOCs were identified among these samples based on the three-dimensional spectrum, which includes the retention time, migration time, and peak intensity (Figure 1a). The diagram illustrates that differences in VOCs were evident at various treatment stages of the tilapia fillets. In Figure 1a, the red vertical line at the abscissa 1.0 is the RIP peak (the reaction ion peak, normalized), and the ordinate represents the retention time of the gas chromatography. Each point on both sides of the RIP peak represents a volatile flavor substance in the wine. The differences in composition and concentration between different samples can be visually displayed according to the presence or absence of a peak (colored point) or the color depth. The darker the color, the higher the content. The drift time range of volatile substances in tilapia fillets was 0.9–2.0 ms, and the retention time was 500–1200 s. Compared to the fresh sample, the red lines of tilapia fillets were darker after pretreatment, drying, and rehydration, and the concentration of volatile flavor substances increased after these processes increased. The processing treatment caused a series of reactions that produced new flavor substances with unique flavor profiles.

The top view (Figure 1b) is shown below for the difference comparison, which is the difference in flavor compounds between the four samples. The total number of compounds in the headspace of each sample is shown by the entire spectrum. Using A (fresh tilapia fillets) as the reference, the signal peak in fresh tilapia fillets was deduced from the other spectra to obtain the difference spectrum among A, B, C, and D (Figure 1b). The blue area indicates that the substance was lower than A in this sample, and the red area indicates that the substance was more abundant than that in the fresh tilapia fillets. Similarly, the darker the color, the greater the difference.

The two-dimensional top view of the volatile substances in different samples of the type and concentration is more intuitive, as shown in Figure 1c, with fresh tilapia samples as a reference for comparison, we can see the changes in volatile flavor substances in tilapia fillets at different stages of processing. The other three groups of samples in the corresponding volatile substances at high and low levels clearly showed that the deeper the red, the higher the corresponding substance concentration compared to the concentration in fresh samples, and the deeper the blue, the opposite effect was observed. The concentration of volatile flavor substances in the processed tilapia fillets was higher than that in the fresh samples. There was no significant difference in the concentration of flavor substances between group B and group C. The number of characteristic peaks of the flavor substances in group D and the signal intensity of the characteristic peaks were weaker than those in groups B and C, which was caused by the loss of water-soluble flavor components during heating and rehydration after drying.

### 3.3. Comparison of the Fingerprints of VOCs in Tilapia Fillets Under Different Processing Stages

**Figure 2:**
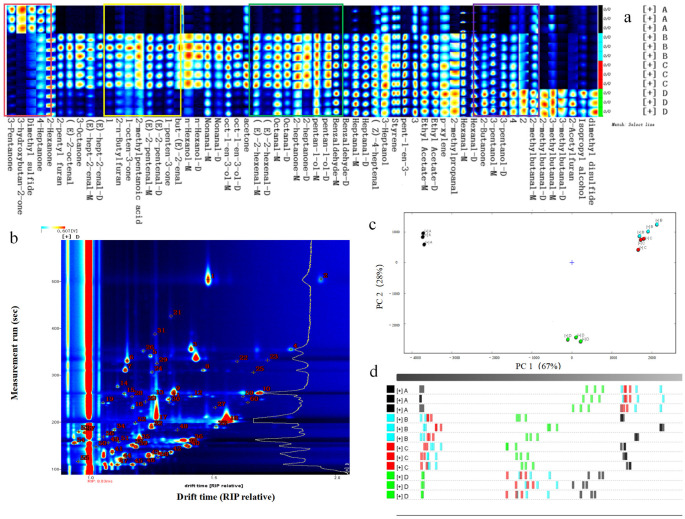
Comparison of VOC fingerprints in tilapia fillets at different processing stages. (**a**) Gallery plot fingerprint of tilapia fillets. (**b**) Library search qualitative analysis. (**c**) PCA of tilapia fillets during processing. (**d**) Euclidean distance diagram of tilapia fillet in processing.

To compare the specific flavor differences in fish fillets at different processing stages, all peaks were selected for the fingerprinting. In Figure 2a, a row represents the composition of the volatile components of a sample, and a column represents the signal peaks of the volatile substances in different samples. The color of the signal peak represents the concentration of each substance in the sample. The complete volatile organic matter information of the four groups of fish and the differences in volatile organic matter between the samples were directly observed. The concentration of flavor substances in fresh fish fillets in this area was relatively higher than that in the other three groups, and the characteristic flavors were 3-pentanone, 3-hydroxy-2-butanone, dimethyl sulfide, and 4-heptanone. The VOC composition and strength of the blanching and pre-freezing groups were relatively similar; however, the levels of 2-pentylfuran, E-2-octenal, 2-butylfuran, E-2-pentenal, E-2-butenal, nonanal, hexanol, and other flavor compounds were higher in the blanching group. The acetone, octanal, pentanol, heptanal, benzaldehyde, and 3-heptanol contents in the pre-freezing group in the C zone were relatively high. The contents of hexanal, 2-butanone, 3-pentanol, 2-methylbutanal, 3-methylbutanal, 2-acetylfuran, and isopropanol in group d were the highest. The ketone concentration in fresh fish was high, and the concentrations of aldehydes and alcohols increased after processing.

**Figure 3:**
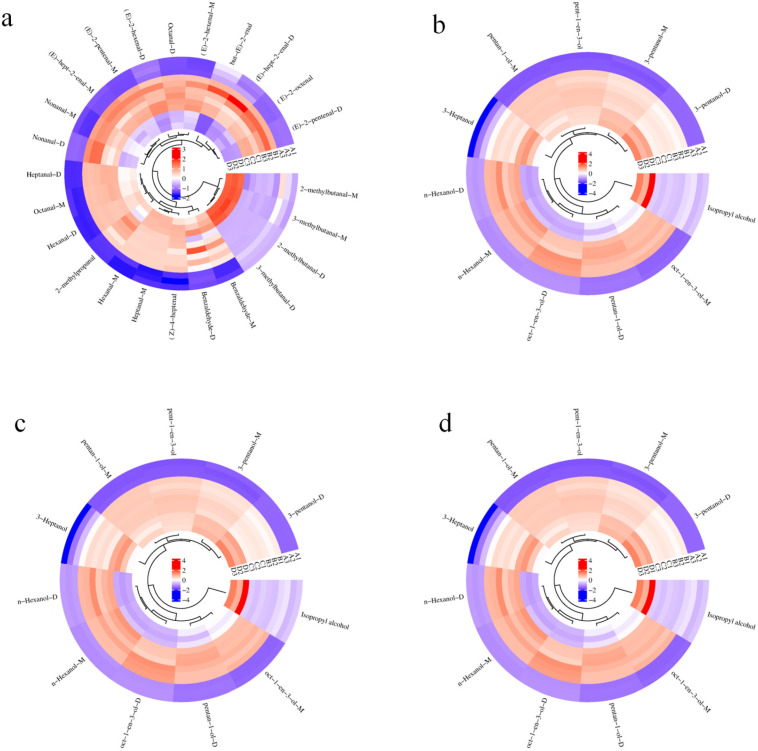
The cluster analysis of volatile compounds in tilapia fillets at different processing stages. (**a**) aldehydes; (**b**) alcohols; (**c**) ketones; (**d**) others.

The volatile compounds in tilapia meat were also characterized in this study. Qualitative analysis of the substances was performed using the NIST and IMS databases integrated into the GC-IMS application software. A total of 60 peaks were identified in the processed tilapia meat (Figure 2b). After a database comparison, 56 volatile components were identified, including monomers and dimers of some volatile flavor substances, including 24 aldehydes, 11 alcohols, 11 ketones, 1 acid, 2 esters, 2 ethers, 1 terpenes, 1 benzene, and 3 heterocycles (Figure 3). With the different changes in volatile content in heated and dried rehydrated fish, multiple flavor compounds together constitute the characteristic flavor of fish.

Concentration and threshold are important factors in determining the volatile compounds in meat flavor [23]. A high threshold has little contribution to meat flavor. Aldehydes are produced by the oxidative degradation of unsaturated fatty acids (UFA). They generally have high thresholds and pleasant odors, such as grassy and fruity ones. Aldehydes are abundant in fish meat and include saturated straight-chain aldehydes such as hexanal, nonanal, octanal, benzaldehyde, and other major flavor compounds. Alcohols are important flavor substances that contribute to plant aromas and earthy smells [24]. Alcohols are formed via sugar metabolism, lipid oxidation, amino acid decarboxylation, and dehydrogenation [25]. The threshold values for these compounds were higher than those of aldehydes, which contribute little to tilapia flavor. The relative concentrations of alcohols observed post fish processing were elevated in the test results, which may be attributable to the heat-induced oxidation of alcohols, as previously reported. Ketones primarily arise as secondary products of fat oxidation, polyunsaturated fatty acid and amino acid degradation, and microbial oxidation. Despite their high thresholds, ketones contribute minimally to the flavor. Fresh fish have a high ketone content, which significantly contributes to their strong odor. Additionally, ketones are crucial intermediates in the formation of certain heterocyclic compounds that can enhance aroma development during processing [26]. Esters are synthesized through various mechanisms, predominantly via acid and alcohol esterification. Esters impart fruity flavors to meat products and significantly influence their aroma.

PCA is a multivariate statistical analysis method that uses multiple variables for linear transformation to select several effective variables [27] and extracts the main features by reducing the dimensions of the data for a linear analysis [28]. As shown in Figure 2c, the cumulative contribution rate of the first two principal components after dimensionality reduction was 95%, which can be used as the main characteristic of fish volatile flavor. The first principal component contribution rate was 67% and the second principal component contribution rate was 28%. Following feature compression, the data retained a more comprehensive set of information, thereby enhancing their ability to distinguish the differences among the original variables. The samples exhibited substantial distances, indicating pronounced differences in their characteristics. The similarity in flavor substances between the blanching and pre-freezing treatments suggests that short-term quick freezing does not significantly impact the flavor profile of tilapia fish. In contrast, a notable distance was observed between the fresh samples and flavor substances after blanching, pre-freezing, and rehydration. The alteration in the odor of heat-treated fish can be attributed to the Maillard reaction and fat oxidation. In contrast, the variation observed in the rehydrated sample was due to the loss of water-soluble flavor components during heating and rehydration of the dried fish. In conjunction with the Euclidean distance analysis presented in Figure 2d, it is evident that samples B and C exhibit the greatest proximity, whereas samples A and D are the most distant from each other. This observation highlights the variations and similarities in volatile organic compound (VOC) composition among the samples.

### 3.4. Analysis of Content Change in Volatile Flavor Compounds

As shown in Table 2 and Figure 3, aldehydes, alcohols, and ketones were the flavor substances in tilapia fillets. The relative ketone content in fresh tilapia was 28.19%, followed by 11.02% in Group C. There was no significant difference in the relative ketone content between groups B and D (7.59% and 7.49%, respectively). This is consistent with previous research, which found that the ketone content in raw fish is high [29] and that the relative ketone content in fish meat decreases after heating [30]. Ketone compounds can enhance the fishy smell, and the decrease in fishy smell after heat treatment is related to the decrease in the relative content of ketones. After simultaneous heating pretreatment, the relative content of aldehydes increased significantly, and their contribution to the overall flavor was higher, promoting tilapia flavor production. The proportion of aldehydes among the total volatile flavor substances was the highest at 48.84% in fresh fish. Groups B, C, and D had 65.84%, 63.13%, and 64.73% content, respectively, which increased by 17%, 14.29%, and 15.89%, respectively. Heat treatment accelerates lipid oxidation in fish meat, increasing the relative content of aldehydes, particularly linear aldehydes such as hexanal, nonanal, octanal, and benzaldehyde. Linear aldehydes in meat mainly originate from the oxidation of unsaturated fatty acids such as oleic, linoleic, and arachidonic acids [31]. C5–C9 aldehydes have unique flavors, such as fragrance, oily fragrance, and oily fragrance [32]. Research has identified that the aldehydes resulting from the oxidation of fish lipids include hexanal, heptanal, and nonanal. Hexanal is linked to a scent reminiscent of grass, whereas octanal has a fresh and slightly burnt smell. Nonanal imparts a fatty aroma to the meat. Benzaldehyde, which may be produced through the metabolism of phenylalanine, is noted for its hay-like flavor and aroma. Unsaturated enals are generally regarded as the oxidation products of polyunsaturated fatty acids and are known for their pleasant aromas [33]. The oxidation products of polyunsaturated fatty acids, specifically nonanal and hexanal, possess high sensory thresholds and are typically regarded as secondary contributors to the flavor profile of processed meat products [34]. Furthermore, the increase in aldehyde content may be associated with the hydrolysis of proteins. Heating pretreatment promotes the hydrolysis of fish proteins to produce free amino acids, which are then oxidatively degraded to form aldehydes [35]. The primary flavor compounds identified in rehydrated dried fish were aldehydes and alcohols. These include nonanal, octanal, benzaldehyde, heptanal, 2-methylbutanal, 1-pentanol, 1-octen-3-ol, and 3-methylbutanol, among others. The relative alcohol content increased significantly (*p* < 0.05), reaching 21.08%, with notable increases in 1-octen-3-ol and 1-pentanol levels (*p* < 0.05). The observed increase in alcohol levels can be primarily attributed to the degradation of proteins and fats in fish during heat treatment. Initially, the 1-octen-3-ol content in fresh tilapia fillets was low; however, the extent of fat oxidation increased following heat treatment, resulting in an increased 1-octen-3-ol content in the fillets after heat treatment. This compound, predominantly oxidized by linoleic acid, imparts a mushroom-like flavor and possesses a low detection threshold, serving as the principal contributor to the overall flavor profile of shiitake mushrooms.

### 3.5. Ready-to-Eat Self-Heating Sauerkraut Fish

**Figure 4:**
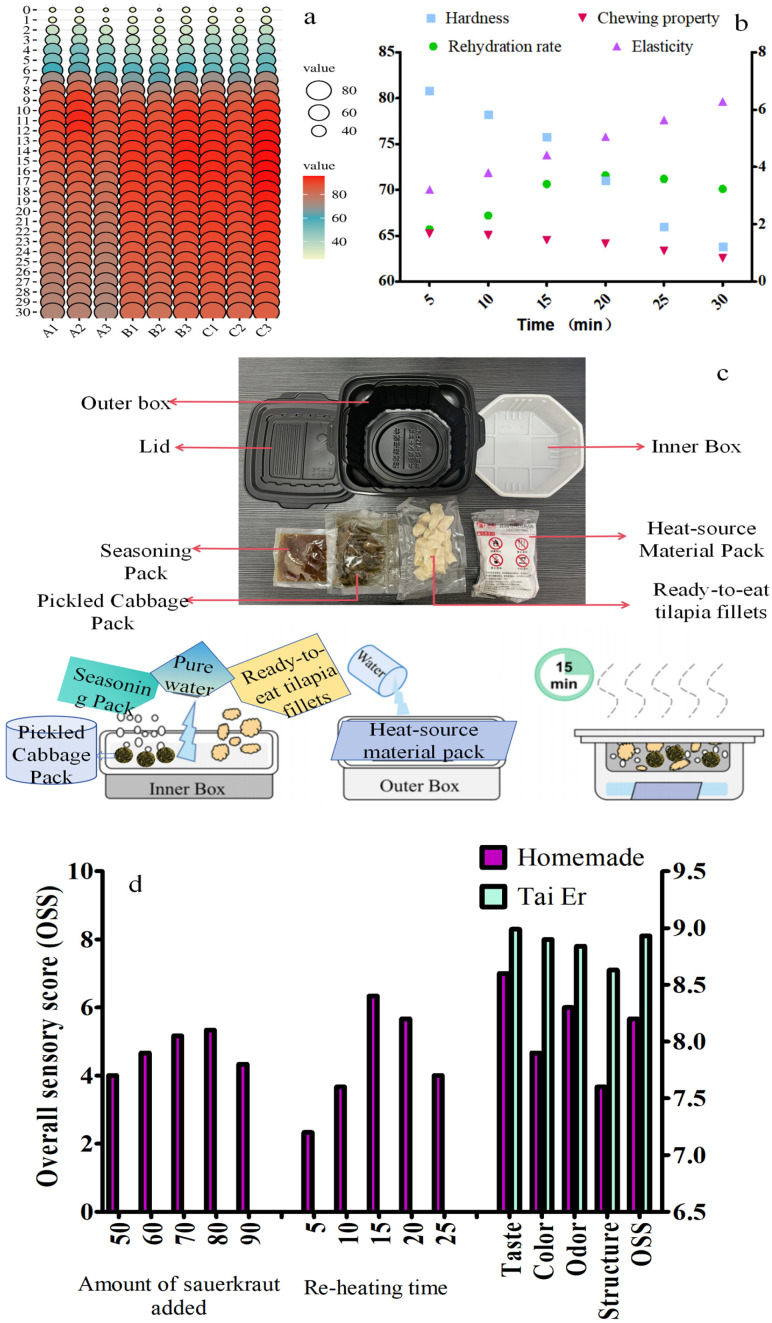
Ready-to-eat, self-heating sauerkraut fish dish. (**a**) Effect of heating package on the center temperature of heated tilapia fillets. A indicates the core temperature of the tilapia fillet, B indicates the water temperature, and C indicates the temperature of the heating source. (**b**) Effect of reheating time on the rehydration rate and texture of tilapia fillets. (**c**) Components of the self-heating sauerkraut fish box and schematic diagram of the preparation process. (**d**) Sensory evaluation comparison of self-heating sauerkraut fish.

Hot meals align with Chinese eating habits, and self-heating foods typically take over ten minutes to heat up, providing benefits of time efficiency, convenience, and speed. As illustrated in Figure 4a, within 10 min, the central temperature of the fish (A), water temperature (B), and heat source temperature (C) were in the rapid heating phase. By the 10-min mark, the central temperature of the fish surpassed the characteristic temperature (85 °C), reaching 91.2 °C at 12 min before transitioning to the cooling stage. The decline rates of the heat source and water temperatures were slightly slower than that of fish temperature. Nevertheless, the central temperature of the fish remained above 70 °C for 30 min, indicating an effective heat retention.

Dried products generally require rehydration before consumption; therefore, rehydration capacity and post-rehydration quality are important indicators for evaluating their quality. The higher the rehydration capacity, the better the quality of the dried product is. As shown in Figure 4b, the rehydration rate at 5 min was 65.69%, and the highest rehydration rate occurred at 20 min, reaching 71.57%. The internal rehydration state of the fish pieces was optimal between 10 and 15 min, meeting the quick-consumption requirements of instant brewing products within 10–20 min. Maintaining a heated state during rehydration helps improve rehydration capacity. Studies have shown that higher rehydration temperatures facilitate tissue expansion, increase space, and enhance molecular diffusion rates, thereby improving the water absorption capacity of samples. The rehydration rate was negatively correlated with the textural indicators. After rehydration with a heating pack, the texture indicators of dried tilapia fillets decreased, and the longer the time, the more significant the texture changes, which affected the fish texture. Figure 4c shows that the hardness and chewiness of fish meat did not change significantly (*p* > 0.05) within 5–10 min, but they significantly decreased (*p* < 0.05) with extended time. After 15–20 min, the hardness of the fish was approximately 70 g, reaching a softened effect, with the hardness decreasing further as time increased. Elasticity and chewiness exhibited similar trends in this study. After reheating, the hardness of the fish meat was between these two values, with lower elasticity, providing a good reference for self-heating fish meat rehydration. During thermal processing, the molecular structure of muscle fibers changes, affecting their texture. During rehydration, longer times and higher temperatures lead to more damaged and degraded muscle fibers, resulting in gradually looser fiber bundles and increased inter-fiber gaps [36], thereby reducing texture indicators. Within 15–20 min, the rehydration rate of the dried fish products was moderate, with insignificant changes in elasticity and chewiness, meeting the rapid rehydration and reheating requirements of self-heating foods.

## 4. Conclusions

Tilapia fillets contain polyunsaturated fatty acids that are affected by temperature, microorganisms, and oxygen during their processing. This promotes fat oxidation, and the TBA value increased from 0.39 ± 0.01 mg/100 g to 0.52 ± 0.01 mg/100 g. Fat oxidation promotes protein oxidation and decomposition, producing alkaline volatile substances that increase pH. Pickling treatment improved the hardness and elasticity of fish after rehydration and effectively maintained a certain edible taste of the freeze-dried fish after rehydration. The effects of different processing stages on volatile flavor substances in tilapia fillets were markedly different. The fishy odor of fresh tilapia fillets decreased after heating and drying processing. The aldehyde content increased after curing and thermal processing, promoting the production of volatile flavor substances in fish and providing a reference for the deep processing of *O. niloticus*. During processing, oxygen and heat treatment promote fat oxidation and protein degradation, facilitating the generation of these aromatic compounds, which leads to fatty acid oxidation, reducing their nutritional value and modifying their taste and smell. Aldehydes, alcohols, and ketones are the main flavor compounds in tilapia. Ketones were present in higher concentrations in fresh fish but decreased after heat treatment, whereas the concentrations of aldehydes and alcohols increased in dried tilapia fillets, confirming that the heating and drying processes improved the flavor of the ready-to-eat fillets. Freeze-dried fish fillets (50 g), pickled cabbage (80 g), and a seasoning packet (50 g) were used, and the heating packet was heated for 15 min. The homemade self-heating pickled fish hot pot exhibited optimal flavor and texture, with overall acceptability comparable to that of commercially available self-heating pickled fish products, providing theoretical guidance for the future development of self-heating tilapia products.

## Figures and Tables

**Table 1:** Effect of different treatments on texture, pH and TBA content of tilapia fillet.

Sample	Hardness/g	Elasticity/mm	Chewing Property/mJ	pH	TBA (mg/100 g)
Fresh tilapia fillet (A)	84.0 ± 1.20 ^c^	2.87 ± 0.18 ^c^	1.58 ± 0.35 ^b^	6.37 ± 0.06 ^b^	0.39 ± 0.01 ^b^
Pickling and blanching tilapia fillet (B)	81.8 ± 1.40 ^d^	3.19 ± 0.28 ^b^	1.59 ± 0.32 ^b^	7.32 ± 0.07 ^a^	0.51 ± 0.03 ^a^
Freeze-dried fillet (C)	96.0 ± 0.70 ^a^	3.70 ± 0.25 ^a^	2.28 ± 0.04 ^a^	7.28 ± 0.0.07 ^a^	0.53 ± 0.03 ^a^
Rehydration tilapia fillet (D)	94.3 ± 0.39 ^b^	3.44 ± 0.23 ^a^	1.61 ± 0.06 ^b^	7.35 ± 0.0.01 ^a^	0.52 ± 0.01 ^a^

Different letters indicate significant difference (*p* < 0.05).

**Table 2:** Qualitative analysis of volatile components in tilapia meat during processing.

Category	NO	Volatile Components	GAS	Rt [s]	Dt [a.u.]	Relative Amount (%)			
						A	B	C	D
Aldehydes	1	Nonanal-M	C_9_H_18_O	503.885	1.48432	5.68 ± 0.33	6.97 ± 0.11	5.77 ± 0.62	4.81 ± 0.27
	2	Nonanal-D	C_9_H_18_O	504.809	1.94227	0.47 ± 0.01	1.54 ± 0.09	0.96 ± 0.04	0.53 ± 0.06
	3	Octanal-M	C_8_H_16_O	358.268	1.41435	1.74 ± 0.12	2.76 ± 0.21	2.84 ± 0.3	2.55 ± 0.05
	4	Octanal-D	C_8_H_16_O	355.032	1.82142	0.38 ± 0.03	1.41 ± 0.11	1.52 ± 0.6	0.69 ± 0.22
	5	Benzaldehyde-M	C_7_H_6_O	310.653	1.14881	1.88 ± 0.25	2.07 ± 0.11	2.10 ± 0.51	2.78 ± 0.95
	6	Benzaldehyde-D	C_7_H_6_O	310.653	1.46365	0.33 ± 0.07	0.54 ± 0.07	0.58 ± 0.11	0.72 ± 0.32
	7	Heptanal-M	C_7_H_14_O	264.426	1.34439	3.60 ± 0.21	3.40 ± 0.19	3.41 ± 0.48	4.20 ± 0.28
	8	Heptanal-D	C_7_H_14_O	263.039	1.69422	0.66 ± 0.08	5.39 ± 1.27	5.59 ± 0.59	4.48 ± 0.71
	9	(Z)-4-heptenal	C_7_H_12_O	260.171	1.14486	0.86 ± 0.05	0.38 ± 0.04	0.39 ± 0.07	0.44 ± 0.06
	10	(E)-2-hexenal-M	C_6_H_10_O	230.717	1.17856	0.62 ± 0.05	0.72 ± 0.12	0.75 ± 0.25	0.46 ± 0.02
	11	Hexanal-M	C_6_H_12_O	202.384	1.27576	12.85 ± 0.88	7.35 ± 0.78	7.36 ± 0.36	8.95 ± 1.42
	12	Hexanal-D	C_6_H_12_O	200.623	1.56347	8.05 ± 0.7	25.90 ± 5.47	25.47 ± 6.31	26.22 ± 4.04
	13	(E)-2-octenal	C_8_H_14_O	425.992	1.33383	0.59 ± 0.01	1.23 ± 0.14	0.93 ± 0.15	0.43 ± 0.05
	14	(E)-hept-2-enal-M	C_7_H_12_O	306.12	1.25718	0.48 ± 0.05	1.36 ± 0.17	1.09 ± 0.13	0.63 ± 0.11
	15	(E)-hept-2-enal-D	C_7_H_12_O	305.827	1.668	0.37 ± 0.04	0.32 ± 0.04	0.22 ± 0.01	0.10 ± 0.02
	16	(E)-2-hexenal-D	C_6_H_10_O	230.243	1.51311	0.29 ± 0.07	0.48 ± 0.01	0.52 ± 0.01	0.13 ± 0.02
	17	(E)-2-pentenal-M	C_5_H_8_O	183.709	1.10507	0.34 ± 0.08	1.09 ± 0.12	0.97 ± 0.05	0.55 ± 0.03
	18	2-methylbutanal-M	C_5_H_10_O	150.641	1.1789	5.75 ± 0.18	1.00 ± 0.08	0.95 ± 0.12	2.26 ± 0.21
	19	2-methylbutanal-D	C_5_H_10_O	151.36	1.39807	0.49 ± 0.08	0.07 ± 0.00	0.06 ± 0.00	1.14 ± 0.17
	20	3-methylbutanal-M	C_5_H_10_O	146.807	1.19531	1.87 ± 0.17	0.30 ± 0.05	0.31 ± 0.06	1.28 ± 0.18
	21	3-methylbutanal-D	C_5_H_10_O	147.046	1.40861	0.44 ± 0.06	0.05 ± 0.00	0.05 ± 0.00	1.03 ± 0.07
	22	(E)-2-pentenal-D	C_5_H_8_O	182.991	1.35939	0.24 ± 0.02	1.08 ± 0.21	0.91 ± 0.16	0.20 ± 0.01
	23	but-(E)-2-enal	C_4_H_6_O	146.807	1.03006	0.83 ± 0.08	0.34 ± 0.03	0.29 ± 0.01	0.09 ± 0.00
	24	2-methylpropanal	C_4_H_8_O	123.902	1.28222	0.05 ± 0.00	0.08 ± 0.00	0.09 ± 0.01	0.07 ± 0.00
Alcohols	25	oct-1-en-3-ol-M	C_8_H_16_O	330.994	1.15676	1.80 ± 0.08	4.29 ± 0.35	4.20 ± 1.62	3.08 ± 0.15
	26	3-Heptanol	C_7_H_16_O	248.966	1.32889	2.10 ± 0.18	0.58 ± 0.03	0.60 ± 0.03	0.68 ± 0.04
	27	oct-1-en-3-ol-D	C_8_H_16_O	329.694	1.60246	0.53 ± 0.02	1.44 ± 0.16	1.11 ± 0.06	0.34 ± 0.01
	28	n-Hexanol-M	C_6_H_14_O	241.702	1.32791	0.25 ± 0.02	0.29 ± 0.04	0.31 ± 0.02	0.10 ± 0.02
	29	pentan-1-ol-M	C_5_H_12_O	189.7	1.25625	1.07 ± 0.06	2.60 ± 0.17	2.57 ± 0.14	2.63 ± 0.09
	30	pentan-1-ol-D	C_5_H_12_O	190.179	1.51644	0.25 ± 0.06	1.88 ± 0.11	2.01 ± 0.09	1.00 ± 0.06
	31	3-pentanol-M	C_5_H_12_O	161.664	1.20703	2.78 ± 0.11	2.32 ± 0.16	2.40 ± 0.21	3.01 ± 0.17
	32	3-pentanol-D	C_5_H_12_O	161.424	1.42151	0.57 ± 0.06	4.23 ± 0.14	4.34 ± 0.57	7.32 ± 1.01
	33	Isopropyl alcohol	C_3_H_8_O	110.863	1.22578	0.86 ± 0.12	0.18 ± 0.01	0.18 ± 0.01	0.65 ± 0.08
	34	pent-1-en-3-ol	C_5_H_10_O	154.248	0.94342	1.01 ± 0.18	2.11 ± 0.12	2.20 ± 0.11	2.25 ± 0.17
	35	n-Hexanol-D	C_6_H_14_O	241.846	1.64601	0.08 ± 0.01	0.16 ± 0.01	0.15 ± 0.01	0.03 ± 0.01
Ketone	36	2-heptanone-M	C_7_H_14_O	255.368	1.2615	1.43 ± 0.18	1.16 ± 0.06	1.20 ± 0.02	0.87 ± 0.01
	37	4-Heptanone	C_7_H_14_O	243.137	1.23111	1.69 ± 0.52	0.18 ± 0.01	0.15 ± 0.01	0.16 ± 0.01
	38	3-Octanone	C_8_H_16_O	332.043	1.72617	0.36 ± 0.01	1.21 ± 0.17	1.02 ± 0.04	0.29 ± 0.01
	39	1-octen-3-one	C_8_H_14_O	325.232	1.27654	0.28 ± 0.11	0.68 ± 0.02	0.55 ± 0.01	0.34 ± 0.02
	40	2-Butanone	C_4_H_8_O	127.876	1.24453	3.62 ± 0.71	1.59 ± 0.09	1.71 ± 0.08	3.35 ± 0.22
	41	acetone	C_3_H_6_O	109.185	1.11679	13.30 ± 1.74	1.79 ± 0.08	5.42 ± 0.31	1.92 ± 0.07
	42	1-penten-3-one	C_5_H_8_O	155.673	1.07928	0.34 ± 0.0	0.31 ± 0.02	0.26 ± 0.02	0.12 ± 0.01
	43	3-Pentanone	C_5_H_10_O	158.069	1.12265	5.42 ± 0.15	0.17 ± 0.01	0.18 ± 0.01	0.20 ± 0.01
	44	2-Hexanone	C_6_H_12_O	197.847	1.19062	1.17 ± 0.29	0.13 ± 0.01	0.12 ± 0.01	0.15 ± 0.01
	45	3-hydroxybutan-2-one	C_4_H_8_O_2_	168.234	1.06025	0.49 ± 0.07	0.01 ± 0.01	0.01 ± 0.01	0.02 ± 0.01
	46	2-heptanone-D	C_7_H_14_O	253.903	1.62724	0.08 ± 0.01	0.36 ± 0.01	0.39 ± 0.01	0.09 ± 0.01
Acid	47	2-methylpentanoic acid	C_6_H_12_O_2_	387.456	1.2694	0.41 ± 0.05	0.71 ± 0.05	0.66 ± 0.09	0.32 ± 0.02
Ester	48	Ethyl Acetate-M	C_4_H_8_O_2_	135.065	1.09686	4.51 ± 0.4	1.57 ± 0.05	1.35 ± 0.13	1.90 ± 0.06
	49	Ethyl Acetate-D	C_4_H_8_O_2_	136.024	1.33361	0.93 ± 0.32	0.92 ± 0.03	0.79 ± 0.08	1.27 ± 0.13
Sulfide	50	Dimethyl sulfide	C_2_H_6_S	115.721	0.95802	1.78 ± 0.17	0.12 ± 0.01	0.21 ± 0.05	0.15 ± 001
	51	dimethyl disulfide	C_2_H_6_S_2_	177.997	0.98139	0.46 ± 0.03	0.09 ± 0.01	0.09 ± 0.01	0.78 ± 0.22
Terpenes	52	Styrene	C_8_H_8_	254.568	1.41702	2.16 ± 0.32	0.44 ± 0.06	0.58 ± 0.01	0.73 ± 0.18
Benzene	53	p-xylene	C_8_H_10_	241.122	1.05932	0.32 ± 0.03	0.07 ± 0.01	0.09 ± 0.01	0.11 ± 0.01
Heterocyclic	54	2-pentyl furan	C_9_H_14_O	342.088	1.25057	0.40 ± 0.02	2.22 ± 0.31	1.71 ± 0.29	0.85 ± 0.22
	55	2-Acetylfuran	C_6_H_6_O_2_	275.538	1.11764	0.29 ± 0.01	0.06 ± 0.01	0.07 ± 0.11	0.43 ± 0.02
	56	2-n-Butylfuran	C_8_H_12_O	254.181	1.1765	0.41 ± 0.03	0.29 ± 0.27	0.23 ± 0.11	0.16 ± 0.01

Note: M: monomer, D: dimer (the same below).

## Data Availability

The original contributions presented in this study are included in the article/Supplementary Materials. Further inquiries can be directed to the corresponding authors.

## Supplementary Materials

The following supporting information can be downloaded at: https://www.mdpi.com/article/10.3390/foods14193293/s1.
