# Supplementary material for: Changes in the Volatile Flavor Compounds and Quality Attributes of Tilapia Fillets Throughout the Drying Process
Source: Foods. 2025 Sep 23;14(19):3293. doi: 10.3390/foods14193293 (PMC12523433; doi:10.3390/foods14193293)
Supplement: Supplementary file 1 [file foods-14-03293-s001.zip › foods-3818344-supplementary.pdf]

Table S1 sensory evaluation standard of ready-to-eat self-heating sauerkraut fish

Indicator	Scoring criteria	Score
Odor	The fish meat has a pronounced aroma and distinct pickled vegetable flavor, with an overall good taste profile<br>The fish meat carries a slight fishy odor with some pickled vegetable flavor, resulting in an average overall taste<br>The fishy odor is relatively strong with insufficient pickled vegetable flavor.	8-10<br>4-7<br>1-3
Taste	The soup has a delicious flavor, with the pickled fish and meat exhibiting good chewiness.<br>The pickled vegetables have an average texture, while the fish meat shows weak chewiness.<br>The meat is overly soft, lacking chewiness in both the pickled vegetables and fish meat.	8-10<br>4-7<br>1-3
Color	The sauerkraut has uniform color, the broth is bright, and the fish meat is evenly colored.<br>The broth is slightly darker in color, and the fish meat has a slight yellowish tint.<br>The broth is pale in color, and the fish meat appears dull in hue.	8-10<br>4-7<br>1-3
Structure	The fish meat is firm with intact tissue structure, and the pickled cabbage maintains good hardness.<br>The tissue structure of the fish meat is somewhat damaged, and the pickled cabbage has softened.<br>The tissue structure of the fish meat is severely damaged, and the pickled cabbage has become overly soft.	8-10<br>4-7<br>1-3
Overall acceptance	Easy to accept, with purchase desire<br>Moderate acceptance, moderate purchase desire<br>Unacceptable, no purchase desire	8-10<br>4-7<br>1-3

Table S2 Volatile components in tilapia fillets under different processing steps

Count	Compound	CAS#	Formula	MW	RI	Rt [sec]	Dt [a.u.]	Comment
1	Nonanal-M	C124196	C9H18O	142.2	1106.2	503.885	1.48432	Monomer
2	Nonanal-D	C124196	C9H18O	142.2	1106.7	504.809	1.94227	Dimer
3	Octanal-M	C124130	C8H16O	128.2	1008.5	358.268	1.41435	Monomer
4	Octanal-D	C124130	C8H16O	128.2	1005.9	355.032	1.82142	Dimer
5	2-pentyl furan	C3777693	C9H14O	138.2	995	342.088	1.25057	
6	oct-1-en-3-ol-M	C3391864	C8H16O	128.2	983	330.994	1.15676	Monomer
7	Benzaldehyde-M	C100527	C7H6O	106.1	960	310.653	1.14881	Monomer
8	Benzaldehyde-D	C100527	C7H6O	106.1	960	310.653	1.46365	Dimer
9	Heptanal-M	C111717	C7H14O	114.2	901.5	264.426	1.34439	Monomer
10	Heptanal-D	C111717	C7H14O	114.2	899.6	263.039	1.69422	Dimer
11	3-Heptanol	C589822	C7H16O	116.2	877.3	248.966	1.32889	
12	Styrene	C100425	C8H8	104.2	886.9	254.568	1.41702	
13	2-heptanone-M	C110430	C7H14O	114.2	888.2	255.368	1.2615	Monomer
14	2-Acetylfuran	C1192627	C6H6O2	110.1	916.5	275.538	1.11764	
15	(Z)-4-heptenal	C6728310	C7H12O	112.2	895.6	260.171	1.14486	
16	(E)-2-hexenal-M	C6728263	C6H10O	98.1	844.5	230.717	1.17856	Monomer
17	Hexanal-M	C66251	C6H12O	100.2	788.1	202.384	1.27576	Monomer
18	Hexanal-D	C66251	C6H12O	100.2	784.3	200.623	1.56347	Dimer
19	p-xylene	C106423	C8H10	106.2	863.5	241.122	1.05932	
20	4-Heptanone	C123193	C7H14O	114.2	867.1	243.137	1.23111	
21	(E)-2-octenal	C2548870	C8H14O	126.2	1058.1	425.992	1.33383	
22	oct-1-en-3-ol-D	C3391864	C8H16O	128.2	981.6	329.694	1.60246	Dimer
23	3-Octanone	C106683	C8H16O	128.2	984.2	332.043	1.72617	
24	(E)-hept-2-enal-M	C18829555	C7H12O	112.2	954.7	306.12	1.25718	Monomer
25	(E)-hept-2-enal-D	C18829555	C7H12O	112.2	954.3	305.827	1.668	Dimer
26	(E)-2-hexenal-D	C6728263	C6H10O	98.1	843.6	230.243	1.51311	Dimer
27	2-n-Butylfuran	C4466244	C8H12O	124.2	886.2	254.181	1.1765	
28	1-octen-3-one	C4312996	C8H14O	126.2	976.7	325.232	1.27654	
29	n-Hexanol-M	C111273	C6H14O	102.2	864.5	241.702	1.32791	Monomer
30	2-methylpentanoic acid	C97610	C6H12O2	116.2	1031	387.456	1.2694	
31	pentan-1-ol-M	C71410	C5H12O	88.1	760.3	189.7	1.25625	Monomer
32	pentan-1-ol-D	C71410	C5H12O	88.1	761.4	190.179	1.51644	Dimer
33	(E)-2-pentenal-M	C1576870	C5H8O	84.1	746.5	183.709	1.10507	Monomer
34	3-pentanol-M	C584021	C5H12O	88.1	691.6	161.664	1.20703	Monomer
35	3-pentanol-D	C584021	C5H12O	88.1	691	161.424	1.42151	Dimer
36	2-methylbutanal-	C96173	C5H10O	86.1	655.4	150.641	1.1789	Monomer

	M							
37	2-methylbutanal-D	C96173	C5H10O	86.1	657.9	151.36	1.39807	Dimer
38	3-methylbutanal-M	C590863	C5H10O	86.1	642	146.807	1.19531	Monomer
39	3-methylbutanal-D	C590863	C5H10O	86.1	642.8	147.046	1.40861	Dimer
40	Ethyl Acetate-M	C141786	C4H8O2	88.1	598.4	135.065	1.09686	Monomer
41	Ethyl Acetate-D	C141786	C4H8O2	88.1	602.1	136.024	1.33361	Dimer
42	2-Butanone	C78933	C4H8O	72.1	569.9	127.876	1.24453	
43	Isopropyl alcohol	C67630	C3H8O	60.1	495.3	110.863	1.22578	
44	acetone	C67641	C3H6O	58.1	487.4	109.185	1.11679	
45	(E)-2-pentenal-D	C1576870	C5H8O	84.1	744.8	182.991	1.35939	Dimer
46	1-penten-3-one	C1629589	C5H8O	84.1	672.6	155.673	1.07928	
47	but-(E)-2-enal	C123739	C4H6O	70.1	642	146.807	1.03006	
48	3-Pentanone	C96220	C5H10O	86.1	680.6	158.069	1.12265	
49	2-Hexanone	C591786	C6H12O	100.2	778.4	197.847	1.19062	
50	pent-1-en-3-ol	C616251	C5H10O	86.1	667.8	154.248	0.94342	
51	Dimethyl sulfide	C75183	C2H6S	62.1	517.7	115.721	0.95802	
52	3-hydroxybutan-2-one	C513860	C4H8O2	88.1	708.7	168.234	1.06025	
53	dimethyl disulfide	C624920	C2H6S2	94.2	733	177.997	0.98139	
54	2-methylpropanal	C78842	C4H8O	72.1	553.4	123.902	1.28222	
55	2-heptanone-D	C110430	C7H14O	114.2	885.8	253.903	1.62724	Dimer
56	n-Hexanol-D	C111273	C6H14O	102.2	864.8	241.846	1.64601	Dimer
